# Baseline serum cortisol as a prognostic biomarker for immune checkpoint inhibitor therapy in advanced gastric cancer

**DOI:** 10.3389/fonc.2026.1728852

**Published:** 2026-05-13

**Authors:** Yufei Zhao, Zhanjun Guo, Jingjing Zhang, Zhidong Zhang

**Affiliations:** 1 Department of Immunology and Rheumatology, The Fourth Hospital of Hebei Medical University, Shijiazhuang, China; 2 The Third Department of Surgery, The Fourth Hospital of Hebei Medical University, Shijiazhuang, China

**Keywords:** Anti-PD-1 antibody, cortisol, gastric cancer, immune checkpoint inhibitors, therapeutic efficacy

## Abstract

**Background:**

Immune checkpoint inhibitors (ICIs) have transformed the treatment of advanced gastric cancer (AGC), but only a subset of patients benefit. Validated prognostic biomarkers for ICI efficacy in AGC remain limited, and the role of endogenous cortisol—an immunosuppressive steroid hormone—in modulating ICI response is unclear.

**Methods:**

This single-center retrospective study enrolled 134 AGC patients who received anti-PD-1 antibody therapy at the Fourth Hospital of Hebei Medical University between January 2019 and September 2023. Baseline serum cortisol and adrenocorticotropic hormone (ACTH) levels were measured via electrochemiluminescence immunoassay. Patients were stratified into low- and high-cortisol groups using a cutoff of 193.95 nmol/L. The primary/secondary endpoints were progression-free survival (PFS), overall survival (OS), and objective response rate (ORR); disease control rate (DCR) was also analyzed. Survival analyses were performed using Kaplan-Meier curves (log-rank test) and Cox proportional hazards models.

**Results:**

The entire cohort had a median PFS of 7.0 months (95% CI: 5.44–8.63) and median OS of 14.5 months (95% CI: 13.59–15.34), with an ORR of 15.7% (95% CI: 9.4–21.9%) and DCR of 79.1% (95% CI: 72.1–86.1%). Baseline serum cortisol showed an AUC of 0.673 (95% CI: 0.58–0.77) for OS prediction with a cutoff of 193.95 nmol/L, while ACTH had an AUC of 0.561 (95% CI: 0.46–0.66) at 25.09 pg/mL. Compared with the high-cortisol group, the low-cortisol group had a significantly higher DCR (93.9% vs. 74.3%, P = 0.016), longer median PFS (10.3 vs. 5.7 months, P = 0.047), and longer median OS (17.4 vs. 13.4 months, P = 0.014). Multivariate analysis confirmed high baseline cortisol as an independent prognostic factor for poorer OS (HR=2.03, 95% CI: 1.20–4.00, P = 0.035) and showed a trend toward significance for shorter PFS (HR=1.64, 95% CI: 1.00–2.69, P = 0.050). In subgroup analysis of 112 microsatellite stability (MSS)/microsatellite instability-low (MSI-L) patients, high cortisol remained independently associated with shorter PFS (HR=1.77, 95% CI: 1.02–3.06, P = 0.042).

**Conclusions:**

Baseline serum cortisol levels are significantly associated with ICI efficacy in AGC patients, with high baseline cortisol correlating with poorer DCR, PFS, and OS. This study identifies baseline serum cortisol as a promising prognostic biomarker for ICI response in AGC, supporting further exploration of stress hormone signaling in immunotherapy optimization.

## Introduction

Gastric cancer (GC) is one of the most prevalent malignancies globally. According to the International Agency for Research on Cancer (IARC), approximately 1.089 million new GC cases were diagnosed worldwide in 2020, ranking it the fourth most common cancer by incidence (1). Despite substantial advances in diagnostic and therapeutic strategies that have improved survival outcomes, early-stage GC is typically asymptomatic, leading to late-stage diagnosis in many patients—this advanced disease is associated with poor prognosis and high recurrence rates. Surgical resection remains the primary treatment for GC, supplemented by multimodal therapies including radiotherapy, chemotherapy, molecular targeted therapy, and immunotherapy (2).

Immune checkpoint inhibitors (ICIs), particularly those targeting the programmed cell death protein 1 (PD-1)/programmed death-ligand 1 (PD-L1) pathway, have revolutionized cancer treatment (3). By blocking this inhibitory signaling axis, ICIs reactivate T cells, enabling them to mount a more robust anti-tumor response (3). Consequently, ICIs have reshaped the therapeutic landscape for numerous advanced cancers and become a cornerstone of disease management—this paradigm shift is particularly notable in advanced gastric cancer (AGC). The landmark CHECKMATE-649 trial ushered in a new era of first-line immunotherapy for AGC (4), while the pivotal KEYNOTE-811 study further validated the efficacy of combining immunotherapy, targeted therapy, and chemotherapy as first-line treatment for human epidermal growth factor receptor 2 (HER2)-positive AGC (5).

Despite these successes, a large proportion of GC patients either fail to respond to ICIs or develop acquired resistance. Currently, validated prognostic biomarkers for ICI response are limited to PD-L1 expression, Epstein-Barr virus (EBV) positivity, high microsatellite instability (MSI-H), tumor mutational burden (TMB), and tumor-infiltrating lymphocytes (TILs) (6). Thus, identifying robust prognostic biomarkers is critical for guiding personalized therapy and optimizing ICI efficacy (6).

Cortisol, an essential steroid hormone synthesized by the adrenal glands, regulates a broad range of transcriptional programs. Its biological effects include modulation of T cell activation, pro-inflammatory cytokine secretion, apoptosis, and immune cell trafficking (7). Glucocorticoids (GCs) and glucocorticoid receptor (GR) signaling have long been recognized for their immunosuppressive roles in immune cells (8). Specifically, GR signaling transactivates the expression of multiple checkpoint receptors and upregulates genes associated with T cell dysfunction following activation (9). While exogenous corticosteroids are widely used to manage ICI-induced immune-related adverse events, the detrimental effects of endogenous GCs on immunotherapy efficacy have been reported in renal cell carcinoma (RCC) (9, 10), pancreatic ductal adenocarcinoma (PDAC) (8), and intracranial cancers (11). However, whether endogenous steroid hormones influence ICI response in GC remains unclear. Therefore, we conducted a retrospective analysis to evaluate the prognostic value of baseline and post-baseline serum cortisol and adrenocorticotropic hormone (ACTH) levels in AGC patients receiving ICI therapy.

## Methods

### Patients

This study included AGC patients who received anti-PD-1 antibody therapy at the Fourth Hospital of Hebei Medical University between January 2019 and September 2023. Patients with a history of prior immunotherapy were excluded. Retrospective data collection included demographic (gender, age) and clinical variables: Eastern Cooperative Oncology Group Performance Status (ECOG PS), combined positive score (CPS), HER2 status, TNM stage, treatment regimen, line of therapy, MSI status, and baseline serum cortisol/ACTH levels. Serum cortisol and ACTH levels are affected by a multitude of factors, including diurnal rhythm, acute stress, chronic comorbidities, liver and renal function, and medication history. To minimize the impact of the aforementioned factors, all blood samples in this study were collected in the early morning (6:00–8:00 AM) under fasting conditions (fasting for more than 8 hours) and in a resting state (no strenuous exercise, no stress stimulation). Serum cortisol and ACTH levels were measured using an electrochemiluminescence immunoassay (ECLIA) kit (Roche Diagnostics, Basel, Switzerland). Baseline levels were defined as measurements obtained before initiating immunotherapy.

To verify consistent circadian phase at sampling, we additionally measured serum melatonin in a subset of 21 patients using the same ECLIA platform. Most melatonin levels were within the normal morning reference range (10–80 pg/mL), confirming standardized and reliable early-morning sampling conditions.The study protocol was approved by the Ethics Committee of the Fourth Hospital of Hebei Medical University (Approval No. 2021136). Informed consent was waived due to the retrospective nature of the study, which utilized only de-identified existing data.

### Treatment and evaluation

Patients received anti-PD-1 antibody therapy every 3 weeks until disease progression. Tumor response was assessed every 2–3 treatment cycles (every 6–9 weeks) via MRI or CT according to RECIST v1.1, continued until radiological progression. The primary and secondary endpoints were progression-free survival (PFS), overall survival (OS), objective response rate (ORR), and disease control rate (DCR).

The definitions of efficacy evaluation terms are as follows: Partial Response (PR): ≥30% decrease in the sum of the longest diameters of target lesions compared with baseline, without new lesions or progression of non-target lesions; Stable Disease (SD): Neither sufficient shrinkage to qualify for PR nor sufficient increase to qualify for progressive disease (PD); Objective Response Rate (ORR): Percentage of patients achieving complete response (CR) or PR; Disease Control Rate (DCR): Percentage of patients achieving CR, PR, or SD.

Two experienced radiologists independently reviewed all images and assessed tumor responses in strict accordance with RECIST v1.1. Discrepancies were resolved by a third senior radiologist to ensure evaluation objectivity and accuracy.

### Statistical analysis

Statistical analyses were performed using SPSS Statistics version 25.0 (IBM Corp., Armonk, NY, USA). The optimal cutoff values of baseline serum cortisol and ACTH were determined by ROC curve analysis with OS as the endpoint, based on the maximum Youden index. Patients were stratified into low- and high-level groups using these thresholds. PFS was defined as the time from anti-PD-1 therapy initiation to disease progression, death, or the end of follow-up. OS was defined as the time from ICI initiation to death from any cause or the last follow-up date.

Categorical variables were analyzed using the Chi-square (χ²) test or Fisher’s exact test. Survival curves were generated using the Kaplan-Meier method, with group differences compared via the log-rank test. Univariate and multivariate survival analyses were conducted using the Cox proportional hazards regression model. Variables with P<0.10 in univariate analysis were entered into multivariate analysis to avoid model overfitting. Correlations between cortisol and melatonin were analyzed using Spearman correlation. A two-sided P-value <0.05 was considered statistically significant.

## Results

### Patient characteristics

A total of 134 AGC patients treated with anti-PD-1 antibodies were enrolled. Treatment regimens included: immunotherapy+chemotherapy (113 patients), immunotherapy+targeted therapy (3 patients), and immunotherapy+targeted therapy + chemotherapy (16 patients). Among all patients, 104 received first-line anti-PD-1-based therapy, 23 received second-line therapy, and 7 received third-line or higher therapy. The baseline clinical characteristics of the cohort and the treatment information of all patients are provided in **Table 1**; **Supplementary Table S1**. ROC curve analyses for cortisol and ACTH are presented in **Supplementary Figure S1**.

**Table 1:** Characteristics of advanced gastric cancer patients with different Cortisol levels.

Covariate	Total No. (%)	Low Cortisol (%)	High Cortisol (%)	*P*
Total	134	33	101	
Age				
< 60	45(33.6)	14(42.4)	31(30.7)	0.215
≥ 60	89(66.4)	19(57.6)	70(69.3)	
Gender				
Male	102(76.1)	27(81.8)	75(74.3)	0.376
Female	32(23.9)	6(18.2)	26(25.7)	
ECOG PS				
0–1	95(70.9)	28(84.8)	68(67.3)	0.053
2–3	39(29.1)	5(15.2)	33(32.7)	
CPS				
< 5	97(72.4)	23(69.7)	74(73.3)	0.690
≥ 5	37(27.6)	10(30.3)	27(26.7)	
MSI status				
MSS/MSI-L	112(83.6)	27(81.8)	85(84.2)	1.000
MSI-H	6(4.5)	1(3.0)	5(4.9)	
HER2 status				
Negative	87(64.9)	18(54.5)	69(68.3)	0.150
Positive	47(35.1)	15(45.5)	32(31.7)	
TNM stage				
III	44(32.8)	12(36.4)	32(31.7)	0.619
IV	90(67.2)	21(63.6)	69(68.3)	
Treatment regimen				
ICI plus chemotherapy	113(85.8)	28(84.8)	85(86.1)	0.618
ICI plus targeted therapy	3(2.2)	0(0.0)	3(3.0)	
ICI plus chemotherapy and targeted therapy	16(12.0)	5(15.2)	11(10.9)	
Treatment lines				
1–2	126(94.0)	31(93.9)	95(94.1)	1.000
≥ 3	8(6.0)	2(6.1)	6(5.9)	

ECOG PS, Eastern Cooperative Oncology Group Performance Status; CPS, Combined Positive Score; MSI status, MSS/MSI-L, microsatellite stability/microsatellite instability-low; MSI-H, high microsatellite instability; HER2, human epidermal growth factor receptor 2; ICI, Immune checkpoint inhibitor.

For the entire cohort, median PFS was 7.0 months (95% CI: 5.44–8.63) and median OS was 14.5 months (95% CI: 13.59–15.34). In total, 21 patients achieved PR and 85 achieved SD (**Table 2**). ORR was 15.7% (95% CI: 9.4–21.9%) and DCR was 79.1% (95% CI: 72.1–86.1%).

**Table 2:** Response to immunotherapy.

Total No.	Low Cortisol group	High Cortisol group
28	2	26
85	26	59
21	5	16
15.7%(95% CI: 9.4-21.9%)	15.2%(95% CI: 2.2-28.1%)	15.8%(95% CI: 8.6-23.1%)
79.1%(95% CI: 72.1-86.1%)	93.9%(95% CI: 85.3-102.5%)	74.3%(95% CI: 65.6-82.9%)

CR, complete response; PR, partial response; SD, stable disease; PD, progressive disease; DCR, disease control rate; ORR, objective response rate.

Using OS as the endpoint, ROC curve analysis yielded an AUC of 0.673 (95% CI: 0.58–0.77) for baseline serum cortisol, with an optimal cutoff value of 193.95 nmol/L determined by the maximum Youden index (0.336; sensitivity=91.4%, specificity=45.2%). For ACTH, the AUC was 0.561 (95% CI: 0.46–0.66), with an optimal cutoff of 25.09 pg/mL (Youden index=0.160; sensitivity=62.9%, specificity=53.1%), indicating weak prognostic value.

### Association between cortisol levels and DCR in AGC patients

As shown in **Table 1**, baseline clinical characteristics (age, gender, ECOG PS, CPS, HER2 status, TNM stage, treatment regimen, line of therapy) were well-balanced between the low- and high-cortisol groups. ORR was comparable between groups (15.2% vs. 15.8%, P = 0.925). In contrast, DCR differed significantly: 93.9% in the low-cortisol group vs. 74.3% in the high-cortisol group (P = 0.016; **Table 2**). These findings suggest that baseline cortisol levels may influence anti-PD-1 therapy efficacy in AGC.

### Association between cortisol levels and PFS/OS in AGC patients

**Figure 1:**
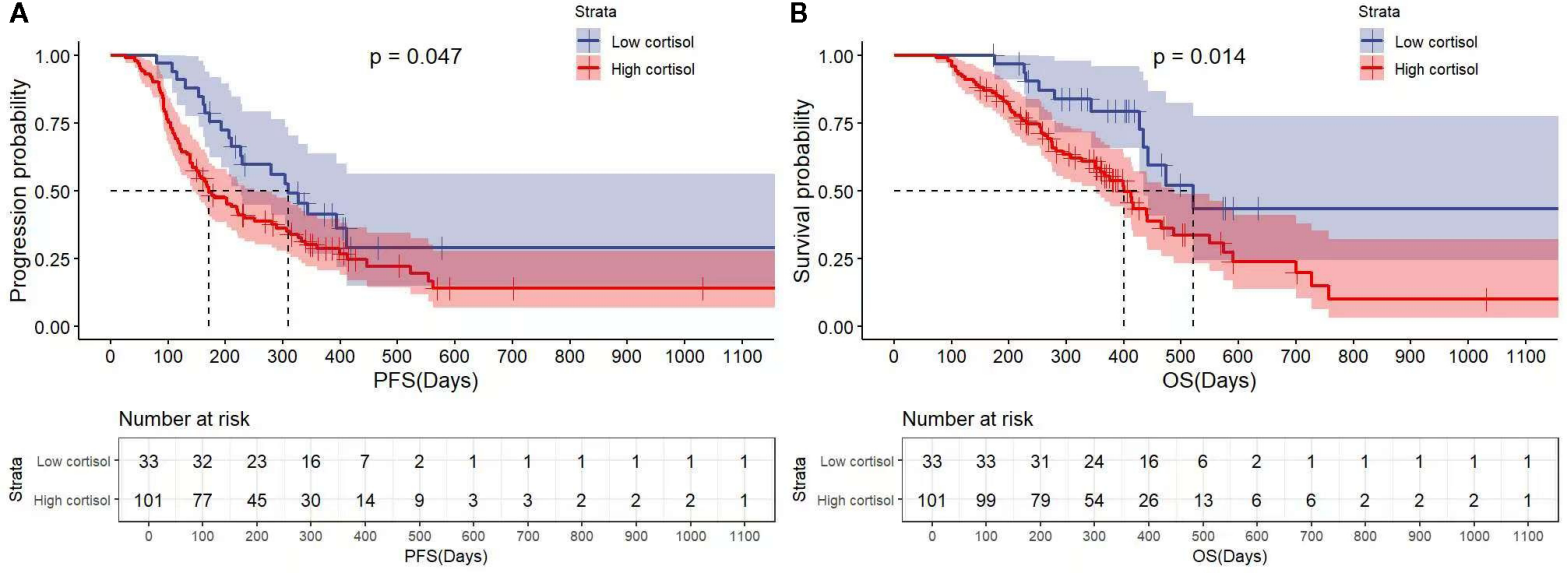
The association of cortisol on the prognosis of advanced gastric cancer patients. **(A)** The Kaplan–Meier curve of progression-free survival (PFS). **(B)** The Kaplan–Meier curve of overall survival (OS).

Kaplan-Meier curves for PFS showed significantly longer median PFS in the low-cortisol group than in the high-cortisol group (10.3 vs. 5.7 months, P = 0.047; **Figure 1A**). Univariate analysis identified gender as a significant predictor of PFS (hazard ratio [HR]=1.59, 95% CI: 1.02–2.49, P = 0.043; **Table 3**). In multivariate analysis including variables with P<0.10 (cortisol and gender), high cortisol showed a marginally significant association with shorter PFS (HR = 1.64, 95% CI: 1.00–2.69, P = 0.050), and female gender remained an independent risk factor (HR = 1.59, 95% CI: 1.01–2.49, P = 0.044; **Table 4**).

**Table 3:** Univariate analyses of PFS and OS.

Covariate	PFS			OS		
	HR	95%CI	*P*	HR	95%CI	*P*
Cortisol						
Low	Reference			Reference		
High	1.641	1.001-2.691	0.049	2.2	1.151-4.204	0.017
ACTH						
Low	Reference			Reference		
High	0.781	0.519-1.174	0.235	0.719	0.441-1.170	0.184
Age						
< 60	Reference			Reference		
≥ 60	1.113	0.722-1.716	0.628	0.962	0.578-1.600	0.880
Gender						
Male	Reference			Reference		
Female	1.589	1.015-2.489	0.043	1.393	0.825-2.352	0.215
ECOG PS						
0–1	Reference			Reference		
2–3	1.367	0.885-2.111	0.159	1.578	0.956-2.604	0.075
CPS						
< 5	Reference			Reference		
≥ 5	0.721	0.453-1.146	0.166	1.212	0.731-2.009	0.455
HER2 status						
Negative	Reference			Reference		
Positive	0.718	0.466-1.109	0.135	0.709	0.417-1.207	0.205
TNM stage						
III	Reference			Reference		
IV	1.321	0.851-2.051	0.215	1.636	0.959-2.792	0.071
Treatment regimen						
ICI plus chemotherapy	Reference			Reference		
ICI plus targeted therapy	1.134	0.278-4.618	0.861	1.492	0.206-10.802	0.692
ICI plus chemotherapy and targeted therapy	1.099	0.243-4.965	0.902	0.99	0.119-8.249	0.993
Treatment lines						
1–2	Reference			Reference		
≥ 3	1.390	0.642-3.011	0.403	0.753	0.299-1.892	0.546

PFS, progression-free survival; OS, overall survival; ECOG PS, Eastern Cooperative Oncology Group Performance Status; CPS, Combined Positive Score; HER2, human epidermal growth factor receptor 2; ICI, Immune checkpoint inhibitor.

**Table 4:** Multivariate analyses of PFS and OS with the Cox proportional hazards model.

	Variables	Total No.	PFS				Variables	Total No.	OS		
			HR	95%CI	*P*				HR	95%CI	*P*
Group						Group					
	Low Cortisol	33	Reference				Low Cortisol	33	Reference		
	High Cortisol	101	1.639	0.999-2.686	0.050		High Cortisol	101	2.027	1.053-3.901	0.035
Gender						ECOG PS					
	Male	102	Reference				0–1	95	Reference		
	Female	32	1.588	1.013-2.490	0.044		2–3	39	1.344	0.805-2.245	0.258
						TNM stage					
							III	44			
							IV	90	1.484	0.862-2.553	0.154

PFS, progression-free survival; OS, overall survival.

The variables included were those with P<0.10 in the univariate analysis.

The low-cortisol group also had significantly longer median OS (17.4 vs. 13.4 months, P = 0.014; **Figure 1B**). Univariate analysis showed trends for ECOG PS (P = 0.075) and TNM stage (P = 0.071). In multivariate analysis including variables with P<0.10 (cortisol, ECOG PS, TNM stage), high baseline cortisol was confirmed as an independent poor prognostic factor for OS (HR = 2.03, 95% CI: 1.21–4.00, P = 0.035; **Table 4**).

### Association between MSI status and ORR in AGC patients

MSI status was assessable in 118 patients, including 112 (94.9%) with microsatellite stability (MSS)/microsatellite instability-low (MSI-L) tumors and 6 (5.1%) with MSI-H tumors. DCR was 80.4% in the MSS/MSI-L group vs. 100.0% in the MSI-H group. ORR was higher in the MSI-H group (50.0% vs. 16.1%), but the difference was not statistically significant (P = 0.117; **Table 5**).

**Table 5:** MSI status associated with immunotherapy response in gastric cancer patients.

Response	Total No.	MSS/MSI-L	MSI-H	*P*
PD	28	22	0	
SD	85	72	2	
PR	21	18	3	
ORR	15.7%(95% CI: 9.4-21.9%)	16.1%(95% CI: 9.2-23%)	60%(95% CI: -8.0-128%)	0.040
DCR	79.1%(95% CI: 72.1-86.1%)	80.4%(95% CI: 72.9-87.8%)	100%(95% CI: 100.0-100.0%)	0.582

CR, complete response; PR, partial response; SD, stable disease; PD, progressive disease; DCR, disease control rate; ORR, objective response rate; MSI status, MSS/MSI-L, microsatellite stability/microsatellite instability-low; MSI-H, high microsatellite instability.

In a subgroup analysis of 112 MSS/MSI-L patients, multivariate analysis confirmed that higher baseline cortisol levels were independently associated with shorter PFS (HR=1.77, 95% CI: 1.02–3.06, P = 0.042). However, no significant association between cortisol levels and OS was observed in this subgroup (P = 0.096).

### Correlation between cortisol and soluble factor

Correlation analysis was performed to explore the relationship between baseline serum cortisol and melatonin. The results showed a negative but not statistically significant correlation between cortisol and melatonin (Spearman r =−0.164, P = 0.482). Notably, most melatonin levels (16/21, 76.2%) were within the normal morning reference range (10–80 pg/mL), confirming that all blood samples were collected under consistent and standardized circadian conditions. Cortisol levels were also within the typical morning physiological range, further supporting the reliability of our sampling protocol. Detailed data are presented in **Supplementary Figure S2**.

## Discussion

AGC is associated with poor prognosis, and conventional chemotherapy has limited efficacy, failing to meet unmet clinical needs. ICIs have transformed AGC treatment: PD-1 inhibitors combined with chemotherapy (for HER2-negative AGC) and PD-1 inhibitors plus trastuzumab and chemotherapy (for HER2-positive AGC) are now established first-line regimens (4, 5). Identifying biomarkers to select patients most likely to benefit from immunotherapy is a key focus of current research. Given the paucity of validated prognostic biomarkers for ICI response in AGC, we conducted this retrospective analysis to evaluate the prognostic significance of baseline serum cortisol levels—and found that these levels have prognostic value for ICI efficacy in AGC. To our knowledge, this is the first study to report an association between cortisol levels and ICI outcomes in AGC patients.

The mechanisms by which cortisol influences ICI efficacy remain incompletely understood, but two key pathways are proposed:

1. **Modulation of chronic inflammation**: Cortisol may indirectly promote GC development and progression by regulating chronic inflammation. In HER2-positive esophagogastric adenocarcinoma, patients with systemic hyperinflammation show poorer responses to immune-targeted combination therapy than those receiving chemotherapy-containing regimens. Metabolomic and cytokine analyses have revealed elevated norepinephrine, cortisol, and IL-6 levels in non-responders to immune-targeted therapy, suggesting that systemic inflammatory stress modulates anti-tumor immune responses (12).
2. **Immunosuppression**: Cortisol exerts direct immunosuppressive effects. Previous studies have shown that GCs play critical roles in regulating T cell development, migration, and function (13). Elevated intratumoral GC levels contribute to TIL dysfunction and promote an exhausted T cell phenotype (9). In RCC, intratumoral expression of 11β-hydroxysteroid dehydrogenase type 1 (HSD11B1)—which converts inactive GCs to active forms—is associated with poor clinical outcomes and an immunosuppressive gene signature. Murine studies have further shown that combining HSD11B1 inhibition with anti-PD-1 therapy increases intratumoral dendritic cell infiltration and improves survival in mice receiving anti-PD-1 treatment, highlighting the role of endogenous GC metabolism in shaping ICI efficacy (10).

The GR is the primary receptor for cortisol and other GCs; upon ligand binding, it regulates gene transcription (14). Under physiological conditions, GC production occurs in the adrenal cortex and is triggered by hypothalamic–pituitary–adrenal (HPA) axis activation (15). Circulating GCs exert systemic effects by binding to GRs expressed in cells throughout the body (8). Cancer patients frequently experience psychological stress, which activates the HPA axis and increases circulating GC levels (16), thereby enhancing GR signaling. In PDAC cells, GR activation transcriptionally upregulates PD-L1 and downregulates major histocompatibility complex class I (MHC-I) expression. In a PDAC mouse model, tumor-specific GR depletion or pharmacological GR inhibition reduced PD-L1 expression, increased MHC-I expression, promoted cytotoxic T cell infiltration and activity, enhanced anti-tumor immunity, and overcome ICI resistance. In PDAC patients, high GR expression correlates with elevated PD-L1 levels, reduced MHC-I expression, and poor survival (8).

Additionally, endogenous GC signaling modulates CD8^+^ T cell differentiation and function in the tumor microenvironment (TME) via the GR (9). Tumor-associated monocyte-macrophages are the primary source of GCs in the TME, influencing CD8^+^ T cell function through local GC synthesis (9). GC signaling suppresses CD8^+^ T cell effector functions and promotes a dysfunctional phenotype by upregulating checkpoint receptors (e.g., PD-1, Tim-3) and the immunosuppressive cytokine IL-10 (17, 18). GC signaling activity is inversely correlated with immune checkpoint blockade (ICB) efficacy: in preclinical models and melanoma patients, high GC signaling is associated with treatment failure (19), while inhibiting GC signaling or synthesis enhances CD8^+^ T cell anti-tumor responses and improves ICB efficacy (20).

In this study, there was no significant difference in Objective Response Rate (ORR) between the high and low cortisol groups (15.2% vs 15.8%, P = 0.925), while a statistically significant difference was observed in Disease Control Rate (DCR) (93.9% vs 74.3%, P = 0.016), and this finding has important clinical implications. The lack of significant difference in ORR suggests that baseline cortisol may not effectively predict early and obvious tumor shrinkage, indicating a limited impact on the degree of tumor regression. In contrast, the significant difference in DCR indicates that cortisol is more closely associated with long-term disease control and tumor stability, and can better reflect the potential for long-term benefits of patients from immunotherapy. Mechanistically, this may be related to cortisol-mediated chronic immunosuppression and regulation of the tumor microenvironment—this regulatory effect is more likely to modulate tumor progression rate and sustain disease stability, rather than inducing rapid tumor regression (21). This also provides a new idea for clinical treatment decisions: for patients with high cortisol levels, focus can be placed on disease control, and treatment strategies can be adjusted if necessary to improve long-term prognosis.

Multivariate analysis showed that female gender was an independent factor associated with shorter PFS, but not an independent predictor of OS, suggesting that its impact may be limited to short-term disease progression rather than long-term survival. This result is consistent with the conclusions of several real-world studies on ICIs treatment for advanced gastric cancer (22), and the underlying mechanisms may involve two key factors: first, gender-related differences in immune function, such as estrogen-mediated regulation of T cell and macrophage function, which in turn affects the efficiency of anti-tumor immune response (23). Second, gender differences in treatment tolerance and adherence.

Regarding the relationship between cortisol and established biomarkers such as PD-L1, MSI, and TMB, the results of this study suggest that serum cortisol may serve as a complementary prognostic biomarker independent of these well-validated classic biomarkers. First, in this study, the ORR of patients with MSI-H tumors was significantly higher than that of patients with MSS/MSI-L tumors, which verifies the classic value of MSI as a predictive biomarker for ICI efficacy in gastric cancer. In the subgroup of MSS/MSI-L patients, who accounted for the main body of the study cohort, cortisol remained significantly associated with PFS, suggesting that even in patients without classic favorable biomarkers, cortisol can still achieve more accurate risk stratification and help screen out populations that may obtain long-term disease control from ICI treatment. Second, in this study, CPS (an indicator related to PD-L1 expression) had no significant correlation with PFS or OS, while cortisol was significantly associated with prognosis, further supporting the potential of cortisol as a complementary biomarker for PD-L1 expression. We propose that combining cortisol with PD-L1, and MSI may establish a more accurate predictive model for immunotherapy benefit, further optimizing the individualized treatment strategy for patients with advanced gastric cancer.

Emerging evidence suggests cortisol may be a viable therapeutic target to enhance ICI efficacy. This hypothesis has been explored using relacorilant, a selective glucocorticoid receptor modulator (SGRM) that acts as a competitive antagonist of cortisol-induced GR signaling (24). In human tumors and immune cells, GR expression correlates positively with PD-L1 expression and T helper 2 (Th2)/regulatory T cell (Treg) infiltration, and negatively with Th1 cell infiltration (7). *In vitro*, cortisol suppresses T cell activation and pro-inflammatory cytokine secretion in human peripheral blood mononuclear cells (PBMCs)—effects reversed by relacorilant. In immunocompetent ovalbumin-expressing tumor models (EG.7, MC38), relacorilant significantly enhanced anti-PD-1 efficacy, improved antigen-specific T cell responses, and modulated systemic TNFα and IL-10 levels (7). These data highlight the broad immunosuppressive role of endogenous cortisol and the therapeutic potential of combining SGRMs with ICIs.

Other ongoing studies evaluate glucocorticoid-induced TNF receptor-associated protein (GITR) agonist antibodies—alone or combined with PD-1 inhibitors—in advanced solid tumors. The combination therapy shows higher response rates in ICI-naive patients (especially those with melanoma), but efficacy in unselected populations is comparable to PD-1 monotherapy (25). Future studies will need to identify potential beneficiaries using combined biomarkers (e.g., GITR expression, TME characteristics).

Based on the results of this study, baseline serum cortisol, as a prognostic biomarker for the efficacy of ICI therapy in advanced gastric cancer, has clear clinical application scenarios, which can be specifically divided into four aspects: First, pre-treatment stratification. By detecting baseline cortisol levels, patients with high cortisol levels who may have poorer disease control and survival can be identified. For such patients, close follow-up and monitoring can be strengthened, or combined treatment strategies (such as combination with SGRMs) can be formulated in advance to improve their treatment outcomes. Second, guidance for treatment decisions. Patients with high cortisol levels may benefit from combined interventions targeting stress hormones or glucocorticoid receptors, providing a basis for the formulation of individualized clinical treatment plans. Third, prognostic assessment. Cortisol detection is a peripheral blood test with the advantages of convenience, minimal invasiveness, and repeatability. It can complement existing biomarkers such as PD-L1 and MSI, further improve the prognostic evaluation system for immunotherapy in patients with advanced gastric cancer, and achieve more accurate individualized management. Fourth, psycho-oncological implications. The results of this study support the importance of stress management and psychological intervention in patients receiving immunotherapy. Alleviating patients’ psychological stress and reducing endogenous cortisol levels may further enhance the efficacy of ICIs, providing a new entry point for the comprehensive management of patients with advanced gastric cancer.

### Study limitations

This study has several limitations. First, it was a single-center retrospective analysis with a relatively small sample size; multicenter, large-cohort prospective studies are needed to validate our findings. Second, this study only detected baseline cortisol levels and did not perform dynamic monitoring of cortisol levels. Although we have supplemented the main confounding factors affecting serum cortisol levels (including diurnal rhythm, acute stress, chronic comorbidities, etc.), and minimized the impact of diurnal variation and acute stress by collecting blood samples on an empty stomach in the early morning (6:00–8:00 AM) and verifying circadian rhythm consistency through melatonin detection, there may still be potential influencing factors that have not been fully covered (such as individual differences in stress response, hidden medication history, etc.), which may have a certain impact on the evaluation of cortisol levels and the stability of the study conclusions. Future studies can further improve the dynamic monitoring program of cortisol levels and comprehensively investigate various potential confounding factors to enhance the reliability of the research results. In addition, while we identified systemic cortisol as a potential biomarker, we did not assess intratumoral lymphocyte infiltration density. Preclinical studies using humanized animal models could provide mechanistic insights into cortisol-mediated TME alterations in GC, particularly regarding immune cell modulation. Furthermore, since TMB levels were not detected in this study, it was impossible to analyze the correlation between cortisol and TMB. Future multicenter, well-designed prospective studies with larger sample sizes are warranted to validate these preliminary findings. Despite these limitations, our findings establish baseline serum cortisol as a promising prognostic biomarker for ICI efficacy in AGC. This discovery supports further exploration of stress hormone signaling pathways as modulators of ICI response across multiple malignancies.

## Conclusions

Baseline serum cortisol levels are significantly associated with ICI efficacy in AGC patients, with high baseline cortisol correlating with poorer DCR, PFS, and OS. This study identifies baseline serum cortisol as a promising prognostic biomarker for ICI response in AGC, supporting further exploration of stress hormone signaling in immunotherapy optimization.

## Data Availability

The original contributions presented in the study are included in the article/**Supplementary Material**. Further inquiries can be directed to the corresponding author.

## Supplementary material

Supplementary Figure S1 ROC curve analysis of baseline serum cortisol and ACTH for predicting overall survival (OS) in advanced gastric cancer patients receiving anti–PD-1 immunotherapy. **(A)** ROC curve of cortisol. The optimal cutoff value was 193.95 nmol/L determined by the Youden index. AUC, 95% CI, sensitivity, and specificity are shown. **(B)** ROC curve of ACTH. The optimal cutoff value was 25.09 pg/mL determined by the Youden index. AUC, 95% CI, sensitivity, and specificity are shown.ROC, receiver operating characteristic; AUC, area under the curve; ACTH, adrenocorticotropic hormone.

Supplementary Figure S2 Correlation between baseline serum cortisol and melatonin in 21 patients with advanced gastric cancer. Blood samples were collected at 6:00–8:00 AM under standardized fasting and resting conditions. The gray shaded area indicates the normal morning reference range of melatonin (10–80 pg/mL). Most melatonin levels (16/21, 76.2%) were within the normal morning range, confirming consistent circadian phase at sampling. Spearman correlation analysis showed a negative but not significant trend (r =−0.164, P = 0.482).

Supplementary Table S1 Treatment information of overall patients.
