# Supplementary figures and images for: Baseline serum cortisol as a prognostic biomarker for immune checkpoint inhibitor therapy in advanced gastric cancer
Source: Front Oncol. 2026 May 13;16:1728852. doi: 10.3389/fonc.2026.1728852 (PMC13212230; doi:10.3389/fonc.2026.1728852)

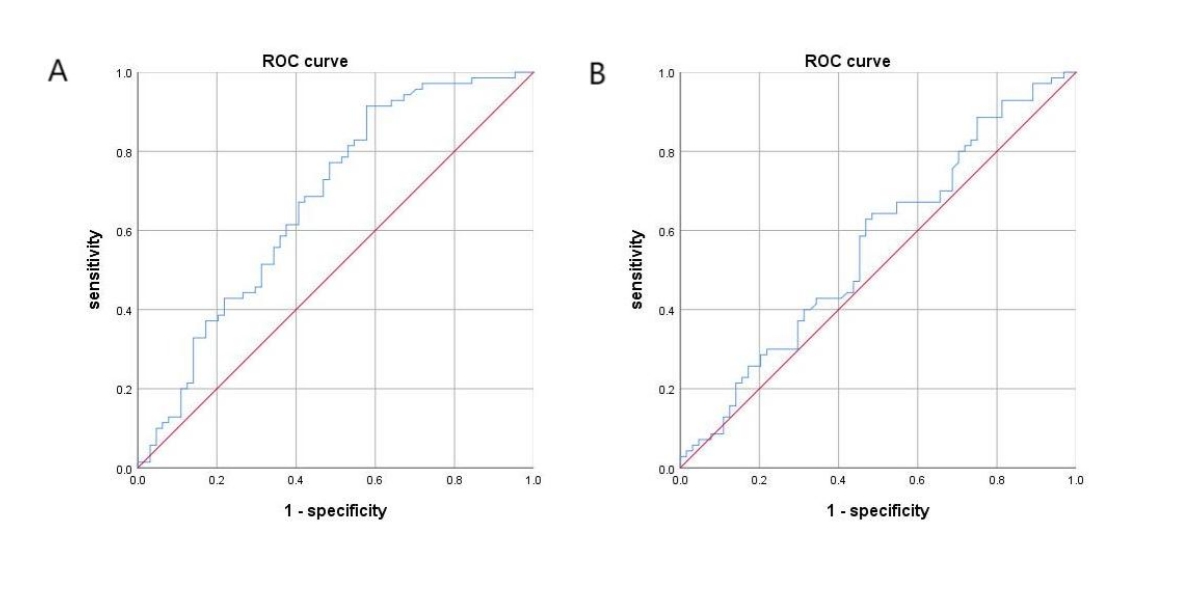

Supplement: Supplementary Figure S1 — ROC curve analysis of baseline serum cortisol and ACTH for predicting overall survival (OS) in advanced gastric cancer patients receiving anti–PD-1 immunotherapy. (A) ROC curve of cortisol. The optimal cutoff value was 193.95 nmol/L determined by the Youden index. AUC, 95% CI, sensitivity, and specificity are shown. (B) ROC curve of ACTH. The optimal cutoff value was 25.09 pg/mL determined by the Youden index. AUC, 95% CI, sensitivity, and specificity are shown.ROC, receiver operating characteristic; AUC, area under the curve; ACTH, adrenocorticotropic hormone. [file Image1.jpeg]

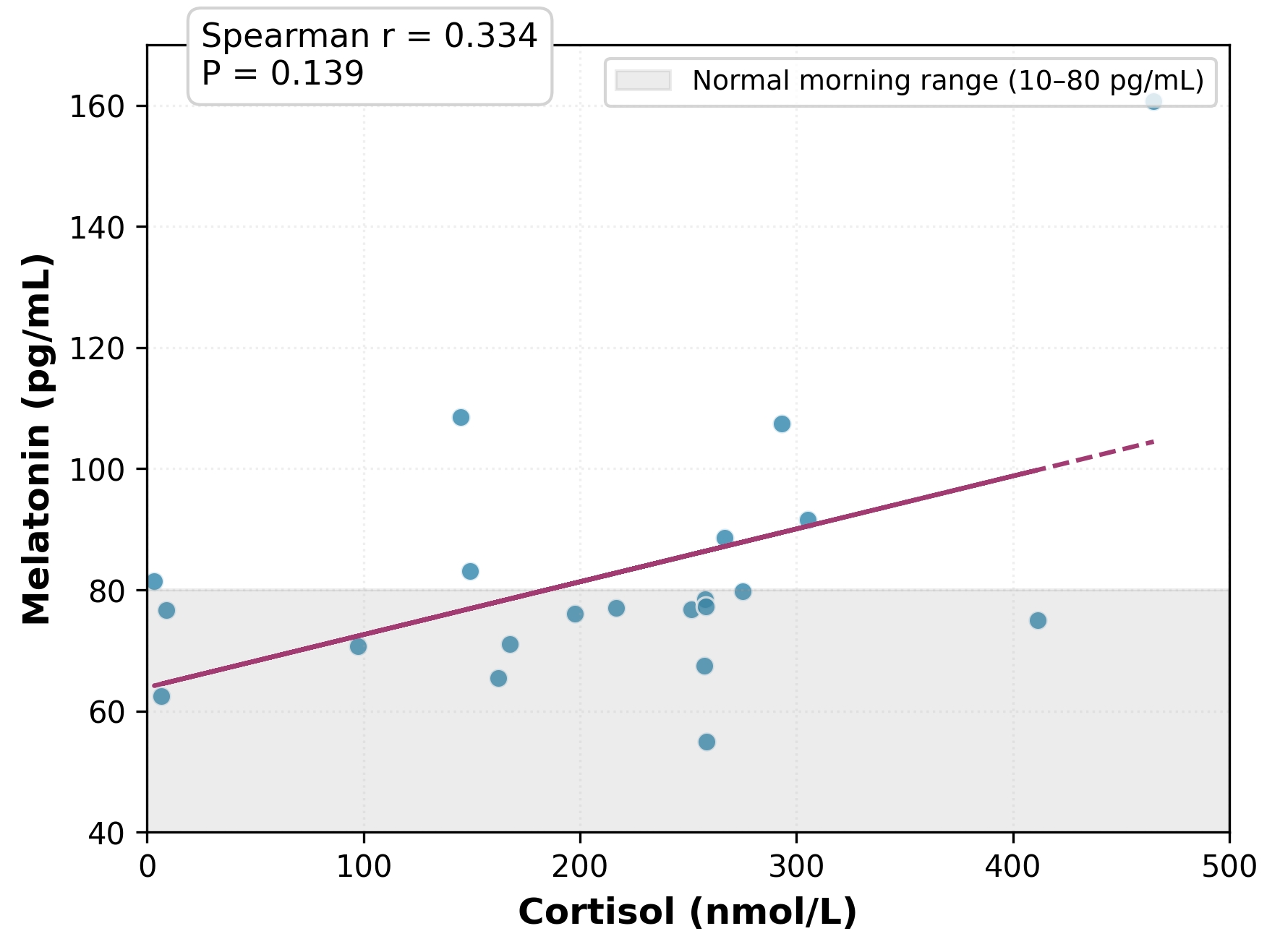

Supplement: Supplementary Figure S2 — Correlation between baseline serum cortisol and melatonin in 21 patients with advanced gastric cancer. Blood samples were collected at 6:00–8:00 AM under standardized fasting and resting conditions. The gray shaded area indicates the normal morning reference range of melatonin (10–80 pg/mL). Most melatonin levels (16/21, 76.2%) were within the normal morning range, confirming consistent circadian phase at sampling. Spearman correlation analysis showed a negative but not significant trend (r =−0.164, P = 0.482). [file Image2.jpeg]
