# Supplementary material for: Baseline serum cortisol as a prognostic biomarker for immune checkpoint inhibitor therapy in advanced gastric cancer
Source: Front Oncol. 2026 May 13;16:1728852. doi: 10.3389/fonc.2026.1728852 (PMC13212230; doi:10.3389/fonc.2026.1728852)
Supplement: Supplementary Table S1 — Treatment information of overall patients. [file Table1.docx]

| **Table S1** Treatment information of overall patients | | | | |
| --- | --- | --- | --- | --- |
| Therapeutic Schedule | | | | |
| Treatment Line | ICIs | Targeted Medicine | Chemotherapeutic Regimen | The number of people |
| 1 | Sintilimab |  | SOX | 19 |
|  |  |  | CAPEOX | 14 |
|  |  |  | FOLFOX(5-FU,leucovorin and oxaliplatin) | 2 |
|  |  |  | Albumin-Bound Paclitaxel | 1 |
|  |  |  | Albumin-Bound Paclitaxel+Oxaliplatin | 3 |
|  |  |  | Albumin-Bound Paclitaxel+S-1 | 3 |
|  |  |  | S-1 | 1 |
|  |  | Trastuzumab | CAPEOX | 1 |
|  |  | Trastuzumab | SOX | 3 |
|  |  | Apatinib | Albumin-Bound Paclitaxel+Oxaliplatin | 1 |
|  |  | Apatinib | Albumin-Bound Paclitaxel | 1 |
|  |  | Apatinib |  | 1 |
|  | Tislelizumab |  | SOX(S-1 and oxaliplatin) | 11 |
|  |  |  | CAPEOX(Capecitabine and Oxaliplatin) | 3 |
|  |  |  | SOX+Albumin-Bound Paclitaxel | 1 |
|  |  |  | Albumin-Bound Paclitaxel+Oxaliplatin | 1 |
|  |  |  | Paclitaxel+S-1 | 1 |
|  |  |  | S-1 | 1 |
|  |  | Disitamab Vedotin | SOX | 1 |
|  |  | Trastuzumab | Capecitabine | 1 |
|  | Camrelizumab |  | CAPEOX | 8 |
|  |  |  | SOX | 3 |
|  |  |  | SOX+Paclitaxel | 1 |
|  |  |  | Albumin-Bound Paclitaxel+S-1 | 1 |
|  |  | Trastuzumab | SOX | 1 |
|  |  | Lenvatinib |  | 1 |
|  | Cadonilimab |  | CAPEOX | 6 |
|  |  |  | SOX | 1 |
|  |  |  | Albumin-Bound Paclitaxel+S-1 | 1 |
|  | Pembrolizumab |  | SOX | 5 |
|  | Nivolumab |  | SOX | 2 |
|  |  |  | CAPEOX | 2 |
|  |  |  | DOS(Docetaxel+Oxaliplatin+S-1) | 1 |
|  | Toripalimab |  | SOX | 1 |
| 2 | Sintilimab |  | SOX | 4 |
|  |  |  | CAPEOX | 4 |
|  |  | Trastuzumab | SOX | 1 |
|  | Pembrolizumab |  | CAPEOX | 2 |
|  |  | Trastuzumab | Albumin-Bound Paclitaxel | 1 |
|  |  | Apatinib | Albumin-Bound Paclitaxel | 1 |
|  | Camrelizumab |  | SOX | 2 |
|  |  |  | CAPEOX | 2 |
|  |  | Apatinib | SOX | 1 |
|  |  | Trastuzumab | Albumin-Bound Paclitaxel | 1 |
|  | Nivolumab |  | SOX | 1 |
|  |  |  | CAPEOX | 1 |
|  | Cadonilimab |  | SOX | 1 |
|  | Tislelizumab |  | Albumin-Bound Paclitaxel+S-1 | 1 |
| 3 | Sintilimab |  | Irinotecan | 3 |
|  |  | Trastuzumab | Docetaxel+S-1 | 1 |
|  |  | Trastuzumab | Albumin-Bound Paclitaxel | 1 |
|  | Camrelizumab | Apatinib |  | 1 |
| 4 | Tislelizumab |  | S-1 | 1 |
